# Supplementary material for: Content-rich biological network constructed by mining PubMed abstracts
Source: BMC Bioinformatics. 2004 Oct 8;5:147. doi: 10.1186/1471-2105-5-147 (PMC528731; doi:10.1186/1471-2105-5-147)
Supplement: Additional File 5 — The original Chilibot query results of the term "long-term potentiation (LTP)" and 22 other terms, limiting the latest references analyzed to the years 1990, 1995, 2000, and 2004. [file 1471-2105-5-147-S5.bz2 › chilibotAdditionalFile5/ltp2004/html/PKA_SYNAPTOTAGMIN.html]

 


 **PKA** and **SYNAPTOTAGMIN** 
  
Found 6 abstracts in PubMed,  **6 abstracts were retrieved and analyzed**.  


---

 Search Google  |
 PDF files only 
|  EDU domain only 

---

**Interactive relationship** (e.g. stimulation, inhibition, etc)

- Mutation of Snapin serine 50 to aspartic acid S50D mimics this effect of  **PKA**  phosphorylation and enhances the association of  **synaptotagmin**  with the soluble N ethylmaleimide sensitive factor attachment protein receptor SNARE complex.  Ref: 11283605 Nat Cell Biol, 2001
- Moreover, the BDNF induced increase of  **synaptotagmin**  was blocked by inhibiting the cAMP protein kinase A  **PKA**  pathway.  Ref: 11483592 J Biol Chem, 2001
- Binding assays in vitro using recombinant proteins confirmed a direct interaction between the two proteins and demonstrated that the  **PKA**  phosphorylated form of CSP binds  **synaptotagmin**  with approximately an order of magnitude lower affinity than the non phosphorylated form.  Ref: 11931641 Biochem J, 2002
- Furthermore, pull down assays using cAMP dependent protein kinase  **PKA**  phosphorylated CSP recovered significantly less  **synaptotagmin** .  Ref: 11931641 Biochem J, 2002
